# A Curiosity-Based Learning Method for Spiking Neural Networks

**DOI:** 10.3389/fncom.2020.00007

**Published:** 2020-02-07

**Authors:** Mengting Shi, Tielin Zhang, Yi Zeng

**Affiliations:** ^1^Research Center for Brain-inspired Intelligence, Institute of Automation, Chinese Academy of Sciences, Beijing, China; ^2^University of Chinese Academy of Sciences, Beijing, China; ^3^Center for Excellence in Brain Science and Intelligence Technology, Chinese Academy of Sciences, Shanghai, China; ^4^National Laboratory of Pattern Recognition, Institute of Automation, Chinese Academy of Sciences, Beijing, China

**Keywords:** curiosity, spiking neural network, novelty, STDP, voltage-driven plasticity-centric SNN

## Abstract

Spiking Neural Networks (SNNs) have shown favorable performance recently. Nonetheless, the time-consuming computation on neuron level and complex optimization limit their real-time application. Curiosity has shown great performance in brain learning, which helps biological brains grasp new knowledge efficiently and actively. Inspired by this leaning mechanism, we propose a curiosity-based SNN (CBSNN) model, which contains four main learning processes. Firstly, the network is trained with biologically plausible plasticity principles to get the novelty estimations of all samples in only one epoch; secondly, the CBSNN begins to repeatedly learn the samples whose novelty estimations exceed the novelty threshold and dynamically update the novelty estimations of samples according to the learning results in five epochs; thirdly, in order to avoid the overfitting of the novel samples and forgetting of the learned samples, CBSNN retrains all samples in one epoch; finally, step two and step three are periodically taken until network convergence. Compared with the state-of-the-art Voltage-driven Plasticity-centric SNN (VPSNN) under standard architecture, our model achieves a higher accuracy of 98.55% with only 54.95% of its computation cost on the MNIST hand-written digit recognition dataset. Similar conclusion can also be found out in other datasets, i.e., Iris, NETtalk, Fashion-MNIST, and CIFAR-10, respectively. More experiments and analysis further prove that such curiosity-based learning theory is helpful in improving the efficiency of SNNs. As far as we know, this is the first practical combination of the curiosity mechanism and SNN, and these improvements will make the realistic application of SNNs possible on more specific tasks within the von Neumann framework.

## 1. Introduction

As neural networks are inspired by the brain at multiple levels and show higher accuracy and wider adaptability compared with algorithms with fixed parameters, they have become one of the important methods for the development of artificial intelligence. The deep neural network (DNN) inspired by the visual cortex has demonstrated its effectiveness in many aspects, such as: visual tasks (He et al., 2017), audio recognition (Audhkhasi et al., 2017), natural language processing (Yogatama et al., 2018), reinforcement learning (Pathak et al., 2017) and etc. However, due to the poor adaptability and interpretability of traditional Artificial Neural Networks (ANNs), more studies have focused on Spiking Neural Networks (SNNs) whose computational units (e.g., Leaky Integrate and Fire Model Gerstner and Kistler, 2002, Hodgkin-Huxley Model, Izhikevich Model Izhikevich, 2003, and Spike Response Model Gerstner, 2001) and plastic learning methods (e.g., Spike-Timing-Dependent Plasticity Dan and Poo, 2004; Frémaux and Gerstner, 2016 and Hebbian learning Song et al., 2000) are more similar to that of the human brain, making it more potential to achieve high levels of cognitive tasks (Maass, 1997; Zenke et al., 2015; Khalil et al., 2017b).

At present, SNNs have been well implemented in some brain regions modeling and cognitive functions simulation, like image classification (Zhang et al., 2018b), working memory maintenance (Zhang et al., 2016), decision-making tasks (Héricé et al., 2016; Zhao et al., 2018), cortical development (Khalil et al., 2017a), contingency perception (Pitti et al., 2009) etc. However, even though the training methods proposed by Zhang et al. (2018a) and Shrestha and Orchard (2018) make SNNs performance comparable to ANNs, they are at the cost of a large amount of time. This is because: (1) the network has a certain degree of overfitting problem when fed with a large number of training samples passively; (2) the SNN's training itself is difficult which needs to process sequential spiking signals; (3) until now, the SNNs are still running on the von Neumann framework instead of the specific designed neural chips, which makes the simulation of neurons inefficient, since the information transformation between CPU and memory usually cost too much time.

Traditional learning methods typically get representations of training data from stationary batches, with little regard to the fact that information becomes incrementally available over time (Parisi et al., 2019). A system with brain-inspired intelligence should be composed of inputs, outputs, and plastic components that change in response to experiences in an environment, and autonomously discover novel adaptive algorithms (Soltoggio et al., 2018).

**Figure 1:**
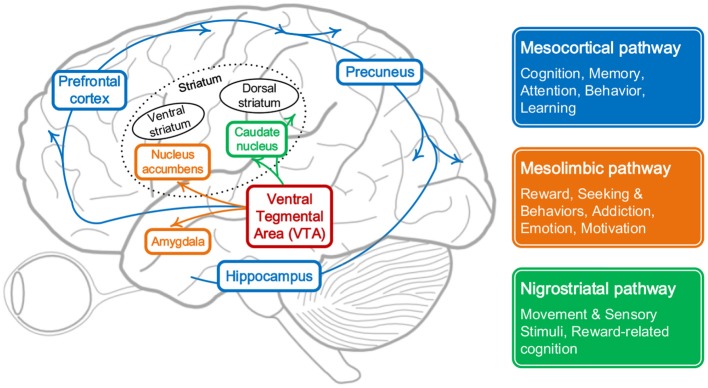
Pathways in the brain that respond to curiosity.

While the curiosity-based learning system in the brain helps us to grasp new knowledge efficiently and actively. From the microscopic point of view, as shown in Figure 1, the continuity of cognitive process in the brain sometimes may be interrupted by some specific unfamiliar or uncertain stimulus, which are mostly from the response of mesolimbic pathway. This pathway is reward pathway (Dreyer, 2010), which connects the ventral tegmental area in the midbrain, to the ventral striatum of the basal ganglia in the forebrain (Ikemoto, 2010) and starts to releases neurotransmitters when facing unfamiliar information, like dopamine, serotonin, and opioid which could regulate characteristics associated with curiosity, like:

**Memory:** The novelty of stimuli can be considered as the result of continual comparison between the current state and previous experiences, which will cover the brain regions related to long-term and short-term memory, e.g., the hippocampus and parahippocampus gyrus. After the comparison, individuals can give a corresponding level of novelty for specific stimuli (Sahay et al., 2011).
**Attention and Learning:** With the limitation of energy and efficiency of the biological system, attention plays a vital role in focusing on the stimuli most important or relevant. Some patients with a degenerative disease, for example, Alzheimer's disease, show bad performance on identifying novel stimuli, during which cells in some brain regions, like hippocampus, don't run well and thus prevent the communication with assessing or rewarding process. Attention is a continuous and gradual learning process during which striatum and precuneus get involved in influencing levels of curiosity in terms of novelty (Zola et al., 2011).
**Motivation:** Curiosity has been described as a desire for learning and knowledge, especially what is unknown (Kang et al., 2009). The idea that dopamine modulates novelty seeking is supported by evidence that novel stimuli excite dopamine neurons and activate brain regions receiving dopaminergic input. In an fMRI study, activation in ventral striatum encoded both standard reward prediction errors and enhanced reward prediction errors during novelty driven exploration (Costa et al., 2014).

Curiosity-based exploration behaviors depend on the estimation of difficulty or novelty of tasks, which are related to one previous learning situation and would update gradually (Faraji et al., 2018). An intuitive paradigm that may enlighten us is designed in Baranes et al. (2014), during which the subjects are allowed to choose games with different levels to get high score freely. After mastering simple skills, people are more likely to repeat hard or novel games frequently instead of spending time on overly comfortable or familiar experiments. This result shows that the difficulty and novelty of tasks have a significant impact on the motivation of exploration.

In this paper, we propose a curiosity-based SNN (CBSNN), and the learning process of this model includes four steps:

Step (1): Before the predefined starting time, the CBSNN is trained with a traditional method to get the novelty estimations of all samples in only one epoch;
Step (2): Once the current iteration time is over the starting time, the CBSNN begins to repeatedly learn the samples whose novelty estimations exceed the novelty threshold and dynamically update the novelty estimations of samples according to the learning results within the retrain interval (we use five epochs later);
Step (3): When the duration of step (2) reaches the retrain interval, the CBSNN retrains all samples once (one epoch) in order to avoid the overfitting of the novel samples and forgetting of the learned samples.
Step (4): The model repeats step (2) and (3) until the algorithm converges.

The MNIST hand-written digit recognition dataset is used to verify the performance of our proposed model. Through a series of experiments, we analyze how the proposed method affects the computation efficiency and learning accuracy of the traditional SNN. By comparing with the state-of-the-art Voltage-driven Plasticity-centric SNN (VPSNN) (Zhang et al., 2018a) under standard architecture, our model achieves higher accuracy of 98.55% with only 54.95% of its computation resources.

## 2. Related Works

Several curiosity-related works have been proposed in different research areas, which include but are not limited to active learning, curriculum learning, sample selection strategies, and reinforcement learning.

Active learning is good at select discriminating samples dynamically from large training data sets and training the model efficiently (Zhou et al., 2017). It pays more attention to some informative and representative data to overcome the labeling bottleneck (Konyushkova et al., 2017). However, the curiosity-based learning strategy dynamically evaluates the difficulty or novelty of the sample and makes a selection based on the current learning situation of the network. The quality of learning results not only depends on the representativeness of the sample itself, but also is more related to the specific learning process.

Bengio et al. (2009) proposed curriculum learning to imitate the characteristics of human learning process, and let the model learn from simple to difficult in multiple stages (Ugur et al., 2007; Chernova and Veloso, 2009). It defines the difficulty level of sample before training, and gives the initial weight distribution. However, in curiosity-based learning process, we tend to predefine an evaluation function, which could be many forms, and let the model adjust dynamically.

Cheng et al. (2018) proposed an active sample selection strategy that reaches state-of-the-art accuracy on visual models ResNet-50, DenseNet-121, and MobileNet-V1, which has a lower computation cost compared with previous networks.

Besides, Schmidhuber (1991a,b) used adaptive “world model” to implement neural controllers and reinforcement learning. The system is “curious” in the sense that it described how the particular algorithm may be augmented by dynamic curiosity and boredom in a natural manner. Pathak et al. (2017) introduced a curiosity assessment module which represents the difference between predicted situation and real situation as an intrinsic reward signal to make agents complete games quicker than just using external reward. Savinov et al. (2019) further uses this strategy to pay more attention to those remarkable situations in order to speed up the completion of tasks by agents and avoid the model falling into local optimum to a certain extent. Inspired by the infants' ability to generate novel structured behaviors in unstructured environments that lack clear extrinsic reward signals, Haber et al. (2018) mathematically modeled this mechanism using a neural network that implements curiosity-driven intrinsic motivation to create a self-supervised agent.

However, most of current studies only discuss the possibility of application and performance improvement of curiosity-based learning mechanism under the traditional ANNs' framework. In this paper, we try to combine this brain-inspired curiosity-based learning mechanism with more biologically plausible SNN to improve its current problems in computation efficiency under the traditional computing system, so that it can be applied more widely in the future.

## 3. Methods

In this section, we will introduce the network architecture (including neuron model and network structure) and the learning process of CBSNN in detail.

### 3.1. The Architecture of CBSNN

The network should be designed with different neuron model, synapse model, network structure or learning method in order to solve different tasks. Diehl and Cook (2015) designed a simple two-layered SNN to achieve MNIST (LeCun, 1998) classification. Zhang et al. (2016) had a recurrent part to store memory and eliminate noise. To have a better performance on complex dataset, Shrestha and Orchard (2018) used feed-forward and back propagation procedure at the same time.

In this paper, we adopt a standard three-layered structure, which is similar to Zhang et al. (2018a) to verify the validity of the curiosity-based mechanism in training SNN.

#### 3.1.1. The Neuron Model

Here we adopt the Leaky integrate-and-fire (LIF) neuron model as the basic processing unit. In the LIF model, the neuron will be regarded as a node. Regardless of the transmission of electrical signals in neurons, the variation of the potential difference *u*(*t*) between inside and outside the membrane at time *t* satisfies the Equation (1).

(1) $$\begin{array}{l} C_{m}\frac{du\left(t\right)}{dt}=-\frac{u\left(t\right)}{Rm}+I\left(t\right) \end{array}$$

where *C*_*m*_ is the membrane capacitance in which m is the abbreviation of membrane, *R*_*m*_ is the membrane resistance, and *I*(*t*) is the weighted sum of all input currents (the weight is usually the connection value *w*_*i, j*_ between neuron *i* and *j*). If we use *V*(*t*) to denote membrane potential, *V*_*L*_ to denote leaky potential, *g*_*L*_ to denote leaky conductance, then we could have Equation (2) which demonstrates the change of membrane potential.

(2) $$\begin{array}{l} C_{m}\frac{dV\left(t\right)}{dt}=-g_{L}\left(V\left(t\right)-V_{L}\right)+I\left(t\right) \end{array}$$

Under the consideration of real brain, we introduce excitatory conductance *g*_*E*_ and excitatory reversal potential *V*_*E*_ and we can have the membrane potential updating Equation (3) based on excitatory conductance.

(3) $$\begin{array}{l} \{\begin{array}{l} \tau_{E}\frac{dg_{E}}{dt}=-g_{E}+\eta \sum_{j\in N_{E}}w_{j,i}\delta_{t}\\ \tau_{m}\frac{dV(t)}{dt}=-(V(t)-V_{L})-\frac{g_{E}}{g_{L}}(V(t)-V_{E}) \end{array} \end{array}$$

**Figure 2:**
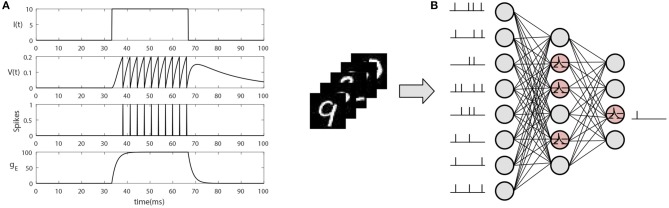
The neuron model and SNN architecture. **(A)** The simulation of a basic LIF neuron. When current *I*(*t*) inputs to the neuron, the membrane potential *V*(*t*) gradually begins to accumulate and the conductance *g*_*E*_ begins to increase. Once the membrane potential reaches the firing threshold, the neuron produces a spike and delivers it to the postsynaptic neuron, while the membrane potential *V*(*t*) returns to the resting state and enters transient refractory period. **(B)** The architecture of a standard SNN. The network is fully connected and the input dataset should be transformed into spike sequence.

When membrane potential integrates up to the firing threshold *V*_*th*_, the neuron produces a spike, and sends it to postsynaptic neurons. After that, the membrane potential is reset to resting state and the neuron enters into refractory time. The simulation results are shown in Figure 2A.

#### 3.1.2. The Network Structure

In this paper, we adopt a standard three layers SNN like (Zhang et al., 2018a). As shown in Figure 2B, the first layer receives sequential signals converted from original dataset; the second layer abstracts the input information using non-linear characteristic; the third layer produces the final classification signals which will be used to transmit error signals and generate novelty estimates with the help of the teacher signals.

### 3.2. The Learning Method of CBSNN

**Figure 3:**
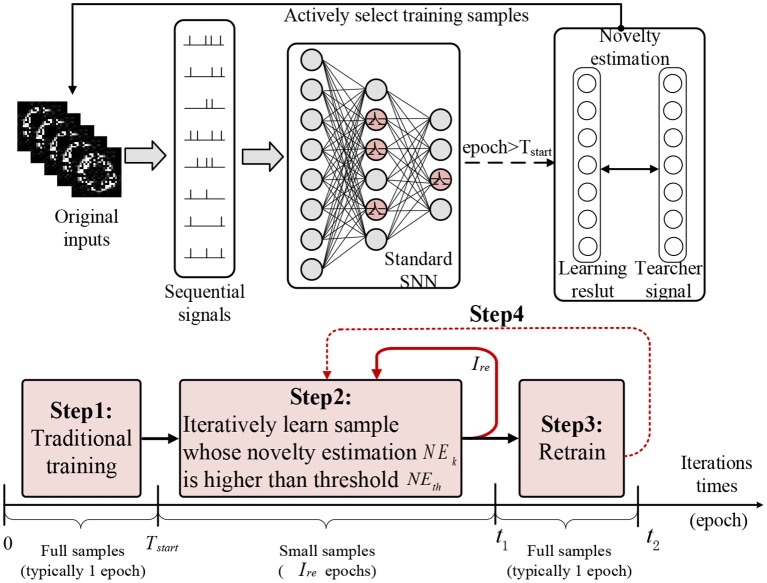
The learning process of CBSNN in spatial and temporal. It processes the sequential input signal and is trained by four steps.

In this section, we will introduce the detailed curiosity-based learning method on SNN (CBSNN). As shown in Figure 3, we first transform the original input data into sequential signals. Then learning process of CBSNN contains four steps: (1) Before the starting time *T*_*start*_ of sample selection (we use one epoch later), the CBSNN is able to train all examples in order to get the novelty estimation of whole dataset; (2) Once the current iteration time is over the predefined starting time *T*_*start*_, the CBSNN begins to repeatedly select the sample whose novelty estimation *NE*_*k*_ exceeds the threshold *NE*_*th*_, and dynamically update the novelty estimations *NE*_*k*_ of samples according to the learning results within the retrain interval *I*_*re*_ (we use 5 epochs later); (3) When the duration of step (2) reaches the retrain interval *I*_*re*_, the CBSNN retrains all data once (one epoch) in order to avoid the overfitting of the novel samples and forgetting of the learned samples; (4) the model repeats step (2) and (3) until the algorithm converges.

#### 3.2.1. Step 1: Traditional Training Before Starting Time *T*_*start*_

At the beginning of learning, we put all data into the SNN before the starting time *T*_*start*_. With sequential signals passing in feed forward, the change of membrane potential $\Delta V_{i}^{FF}$ of neuron *i* and excitatory conductance Δ*g*_*E*_ in this stage are first updated by Equation (3). Then according to the equilibrium tuning which is one of the efficient ways to solve the non-differential problem of SNN (Scellier and Bengio, 2017), the membrane potential is changed again by Equation (4) (Zhang et al., 2018a).

(4) $$\begin{array}{l} \Delta V_{i}^{ES}=-\eta_{i}\frac{V_{i}-(\sum_{j}^{N}w_{j,i}V_{j}-\sum_{j}^{N}V_{th,i})}{-(V_{i}-V_{L})-\frac{g_{E}}{g_{L}}(V_{i}-V_{E})} \end{array}$$

Combining the result of these two stages shown in Equation (5), we get the final change of membrane potential of neuron *i* in an unsupervised way.

(5) $$\begin{array}{l} \Delta V_{i}=\frac{t}{T}\Delta V_{i}^{FF}+\left(1-\frac{t}{T}\right)\Delta V_{i}^{ES} \end{array}$$

In order to let the model have a better performance and calculate the novelty estimation of samples, we introduce the teacher signal *V*_*T*_. By optimizing the loss function $C=\overset{L_{3}}{\underset{i=1}{\sum }}{\left(V_{i}-V_{T}\right)}^{2}$, the membrane potentials of final layer are changed as Equation (6) in supervised way.

(6) $$\begin{array}{l} dV_{i}=-\eta^{c}\left(V_{i}-V_{T}\right) \end{array}$$

#### 3.2.2. Step 2: Curiosity Learning Based on Novelty Estimation *NE* and Novelty Threshold *NE*_*th*_

The weights among these three layers can be updated with multiform Spike-Timing-Dependent Plasticity (STDP) (Dan and Poo, 2004). Here we use a simple but effective one: bi-STDP. Once the current iteration is up to the predefined starting time *T*_*start*_, the weights of the model will be passively changed through Equation (7) ($V_{i}^{\prime }$, is the derivation of *V*_*i*_).

(7) $$\begin{array}{l} \Delta w_{j,i}\propto V_{j}V_{i}^{\prime } \end{array}$$

After the updating of weights, we should get the assessment of samples' learning situation in order to provide an efficient way to select appropriate samples to train in the next iterations. According to the curiosity theory, humans tend to explore novel and difficult problems rather than spend time on general and simple samples in the learning process. Inspired by this, we define the novelty estimation *NE* for samples during the SNN learning process. As shown in Equation (8), instead of error rate, we adopt a similarity evaluation method: the cosine distance between training outputs *V*_*k*_ and the corresponding teacher signals *V*_*T*_, to get more concrete novelty estimation of the sample *k*.

(8) $$\begin{array}{l} NE_{k}\left(V_{k},V_{T}\right)=1-cos<V_{k},V_{T}>=1-\frac{V_{k}\cdot V_{T}}{\left\|V_{k}\|\|V_{T}\right\|} \end{array}$$

According to the novelty estimation *NE*_*k*_ of the sample *k* and predefined novelty threshold *NE*_*th*_, we can obtain the sample selection strategy as shown in Equation (9). And when *S*(*k*) equals to 1, the *kth* sample is selected. Then, the CBSNN repeatedly trains the samples whose novelty estimations exceed the threshold *NE*_*th*_ and temporarily ignores the simple samples which have already learned well, and dynamically updates the novelty estimations of samples using Equation (8) based on the learning results.

(9) $$\begin{array}{l} S(k)=\{\begin{array}{cc} 1 & NE_{k}\geq NE_{th}\\ 0 & NE_{k}<NE_{th} \end{array} \end{array}$$

#### 3.2.3. Step 3: Anti Overfitting and Catastrophic Forgetting Based on Retrain Interval *I*_*re*_

If the model only selects novel samples to train in every iteration, it is inevitable to cause overfitting of novel samples and forgetting of simple samples. So the model needs to review the whole dataset (novel and non-novel) once the duration of dynamic training of step (2) reaches the retrain interval *I*_*re*_ (Kirkpatrick et al., 2017). The more detailed learning process of CBSNN is shown in Algorithm 1.

**Algorithm 1:** The learning process of CBSNN

1. Initialize *T*_*start*_ = 1, *NE*_*th*_ = 0.05, *I*_*re*_ = 5 and other parameters of the network.
2. Load dataset (*X*, *Y*)
3. Start training procedure
*X*_*s*_ ← *X*, *Y*_*s*_ ← *Y*, *e*_0_ ← *T*_*start*_
**for** every epoch *e* **do**
**if** *e* ⩽ *T*_*start*_ **then**………………………………step 1 ▿
SNNTraining(*X*_*s*_, *Y*_*s*_, fullsample=True)
**end if**
**if** *e* > *T*_*start*_ **then**
**if** *e* − *e*_0_! = *I*_*re*_ **then**…………………………step 2 ▿
*NE*(*V*_*k*_, *V*_*T*_) ← 1 − *cos* < *V*_*k*_, *V*_*T*_ > Equation (8)
(*X*_*s*_, *Y*_*s*_) ← *select*(*NE*(*V*_*k*_, *V*_*T*_) ≥ *NE*_*th*_)
Equation (9)
SNNTraining(*X*_*s*_, *Y*_*s*_, fullsample=False)
**else**…………………………………………step 3 ▿
*X*_*s*_ ← *X*, *Y*_*s*_ ← *Y*, *e*_0_ ← *e*
SNNTraining(*X*_*s*_, *Y*_*s*_, fullsample=True)
**end if**
**end if**
**end for**
4. Start testing procedure
**Function:** SNNTraining(*X*_*s*_, *Y*_*s*_, fullsample=True)
**for** every batch *b* **do**
**for** every differential time Δ*t* **do**
$\Delta V_{i,b}^{FF}\leftarrow$ *Feed forward* (*X*_*s, b*_) by Equation (3)
$\Delta V_{i,b}^{ES}\leftarrow$ *Equilibrium state* (*V*_*i, b*_) by Equation (4)
$\Delta V_{i,b}\leftarrow \frac{t}{T}\Delta V_{i,b}^{FF}+\left(1-\frac{t}{T}\right)\Delta V_{i,b}^{ES}$ Equation (5)
$\Delta V_{i,b}^{\prime }$ *supervised tuning* (*V*_*i, b*_) by Equation (6)
**end for**
**end for**
Passively update weights by Equation (7)

## 4. Experiments

In this section, we verify the effectiveness of CBSNN and analyze how the proposed method affects the computation efficiency and learning accuracy of the traditional SNN. And all of our experimental results are based on MNIST.

### 4.1. Hyperparameter Configuration on MNIST

It is hard to get the best values with an exhaustive search for the limitation of computation cost, especially when given a large network. Here we firstly get the best hyperparameters from a smaller network and then apply them to a larger network. The hyperparameters includes *NE*_*th*_, retrain interval *I*_*re*_ and *T*_*start*_. We set an initial CBSNN which is VPSNN with 200 hidden neurons, novelty threshold *NE*_*th*_ = 0.05, retrain interval *I*_*re*_ = 5, starting time *T*_*start*_ = 1. And the following analysis only changes the corresponding parameters on the basis of this initial CBSNN. The specific computation efficiency is calculated by the ratio of the computational time cost of CBSNN and VPSNN.

#### 4.1.1. Starting Time *T*_*start*_

**Figure 4:**
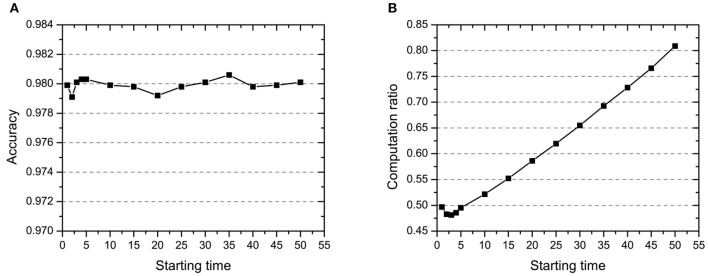
The effect of starting time *T*_*start*_ on **(A)** accuracy and **(B)** computation ratio.

Before the starting time *T*_*start*_, the network is trained to get the novelty estimate of all samples based on the teacher's signal in few epochs. After that, the network starts to select samples through the novelty threshold and dynamically updates their novelty estimation in every iteration. From Figure 4A, we can see that the starting time has little effect on the accuracy which is basically stable at about 0.98. While, the computation in Figure 4B has significant proportional increase when starting time changes from 5 to 50. That means the starting time is robust to the accuracy and can greatly improve the computation efficiency with small value. The result reveals that there is no use to pour the whole data set into the network during all iterations. The earlier the sampling based on novelty estimation, the higher the computation efficiency of the model. And this is the main motivation that we set a small starting time (one epoch) as the hyperparameter in step one of the curiosity-based learning process.

#### 4.1.2. Novelty Threshold *NE*_*th*_

**Figure 5:**
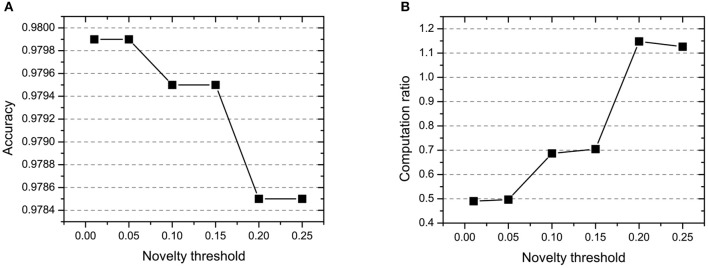
The effect of novelty threshold *NE*_*th*_ on **(A)** accuracy and **(B)** computation ratio.

In CBSNN, the novelty threshold *NE*_*th*_ determines the volume of the difficult samples which will be repeatedly learned. The definition of novelty threshold depends on the difficulty and scale of tasks. In step 1 and step 3, the *NE*_*th*_ is 0 because we need to learn the features of all samples, and in step 2, we set the *NE*_*th*_ changing from 0.01 to 0.25 for getting the best threshold which could balance the learning accuracy and computation cost. As shown in Figure 5, the larger novelty threshold, the more computation ratio and the lower accuracy we will get. The reason is that the large novelty threshold causes the network to repeatedly learn many simple samples, which leads to overfitting of simple samples and wastes a large amount of computation. Especially, the performance of CBSNN is better than VPSNN in all conditions which shows the effectiveness of the novelty threshold.

#### 4.1.3. Retrain Interval *I*_*re*_

**Figure 6:**
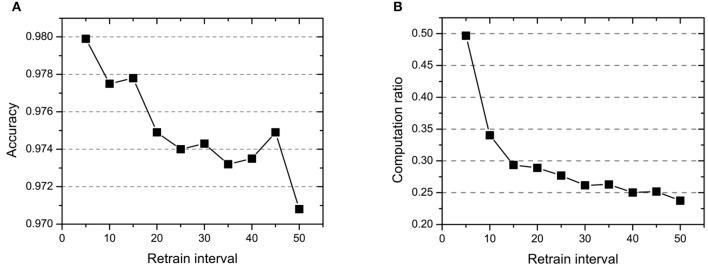
The effect of retrain interval *I*_*re*_ on **(A)** accuracy and **(B)** computation ratio.

The retrain interval affects the frequency of retraining of all samples. As shown in Figure 6, the retrain interval changes from five epochs to 50 epochs and is inversely proportional to accuracy and computation ratio. This parameter leads to a significant decrease in computation (30%), while the accuracy has a little decrease (1%). Especially, even if the retrain interval equals to 50 epochs (only retrain all samples 2–3 times during the whole training), the model can still reach 0.9708 accuracy with 23.75% computation.

To sum up, all parameters have a significant contribution to improving accuracy and reducing computation ratio. And the combination of these parameters is a complex nonlinear relationship. When the CBSNN has comparable accuracy with the VPSNN, the increase in the starting time and the novelty threshold results in a rise in the amount of computation, and the increase in the retrain interval brings about a computation saving.

### 4.2. Performance of CBSNN on MNIST

The CBSNN has three main parameters: starting time *T*_*start*_, novelty threshold *NE*_*th*_ and retrain interval *I*_*re*_. Combining with the above parameter analysis, we finally obtain a set of parameters with high accuracy and low computational complexity that is starting time *T*_*start*_ = 1 epoch, novelty threshold *NE*_*th*_ = 0.05, and retrain interval *I*_*re*_ = 5 epochs.

Based on the optimal combination of parameters, we compare several different strategies to outstand the effectiveness of our approach further. Table 1 shows the comparisons of accuracy and corresponding computation ratio based on the different strategies. The comparisons from the first row to fourth row is based on the 200 hidden neurons. Our best result is 0.16% higher than the original VPSNN, and only requires 49.68% computation. When original VPSNN trained with all data costs around 49% computation, the accuracy of it decreases to 0.9773. When VPSNN is trained by 49% random data, there is a drop of 0.69% in accuracy compared with CBSNN which means curiosity-based learning method is important to actively dig difficult samples. The fifth row of Table 1 shows the best accuracy of VPSNN (0.9852) with 4,500 hidden neurons while our proposed CBSNN achieves 0.9855 accuracy with only 54.95% of VPSNN computation.

**Table 1:** The comparison of different strategies.

**Strategy**	**Hidden neurons**	**Accuracy**	**Computation ratio (%)**
VPSNN	200	0.9783	100
VPSNN with 49% computation	200	0.9773	49.74
VPSNN with 49% random data	200	0.9730	49.43
**CBSNN (ours)**	200	**0.9799**	**49.68**
VPSNN (best) (Zhang et al., 2018a)	4500	0.9852	100
**CBSNN (our best)**	3000	**0.9855**	**54.95**

*First part is the comparison of classfication accuracy and computation ratio among different sample selecting strategies under 200 hidden neurons. The computation ratio baseline is the VPSNN trained with whole MNIST dataset. The second part is the comparison of the best results of VPSNN and CBSNN. The results of our proposed method in this paper are highlighted in bold*.

**Figure 7:**
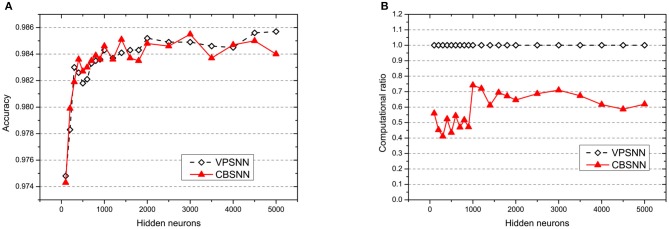
The results of **(A)** accuracy and **(B)** computation ratio of VPSNN and CBSNN under different network structures. The solid red triangle line represents CBSNN, and the dotted white diamond line represents VPSNN. In **(B)**, the baseline of computation ratio under each structure is the time consuming of VPSNN.

In order to compare with VPSNN in large-scaled architecture, we set the hidden neurons from 100 to 5,000 and keep starting time *T*_*start*_ = 1 epoch, novelty threshold *NE*_*th*_ = 0.05 and retrain interval *I*_*re*_ = 5 epochs. As shown in Figure 7, CBSNN can basically reproduce VPSNN accuracy at every level of the number of hidden neurons, and extremely save the computation (at least 25%). The best accuracy of CBSNN is 0.9855 with 3,000 neurons which is better than VPSNN.

### 4.3. Analysis of the Inner State of CBSNN on MNIST

The hidden layer and output layer represent highly abstract features which could account for the specific learning situation. t-SNE (Maaten and Hinton, 2008) can decrease high-dimensional data into two or three dimensions and maintain the relationship of original data as much as possible. Here we use it to observe the change of relationship among all samples when passing these two layers and analyze why our proposed method works during the learning process.

As shown in Table 2, every point represents a sample and different colors represent different classes. We set two different sets of parameters for CBSNN. In first group, we have 400 hidden neurons, starting time *T*_*start*_ = 1 epoch, novelty threshold *NE*_*th*_ = 0.05 and retrain interval *I*_*re*_ = 5 epochs. After the first step of CBSNN, the computation ratio is 0.92%, the accuracy rate is 0.9540 and we get the initial novelty estimation of all samples. At this time, we can see that most of the samples have already formed different clusters but some of them are still very discrete and could not be well classified. Then the CBSNN begins to dig out the difficult samples. During this learning process, those discrete and difficult samples are gradually being better learned. Finally, all samples can be well classified and our model reaches 0.9836 accuracy while computation is 40.43%. At this time, the distance among different clusters is larger, and the distance among samples in each cluster is closer. While the original VPSNN with 400 hidden neurons only has 0.9826 accuracy. In second group, we have 400 hidden neurons, starting time *T*_*start*_ = 1 epoch, novelty threshold *NE*_*th*_ = 0.25 and retrain interval *I*_*re*_ = 50 epochs. Under this configuration, the CBSNN could learn faster but have lower accuracy. Compared with the first group, there are more points being misclassified in every iteration time, and the distance between different classes is closer. When the iteration time is 150, the second group only accounts for 20.31% of the computation ratio, but has 0.9753 accuracy which is lower than that of the first group when it reaches 14.97% computation ratio. Experiments show that the optimized and balanced parameter combination can improve the learning rate and accuracy, and also demonstrate the effectiveness of CBSNN.

**Table 2:** The t-SNE visualization of CBSNN learning process with two sets of different parameters.

**Iteration time**	**1**	**50**	**100**	**150**
Parameter setting one: hidden neurons = 400, *T*_*start*_ = 1, *NE*_*th*_ = 0.05, *I*_*re*_ = 5				
Hidden layer	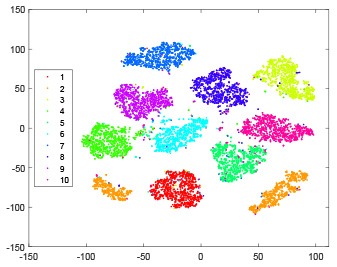	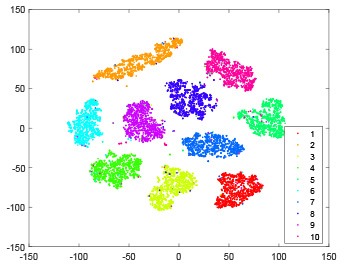	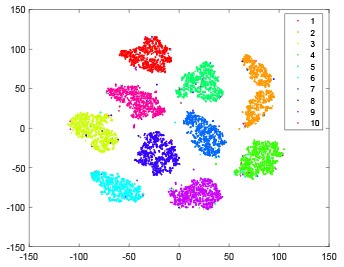	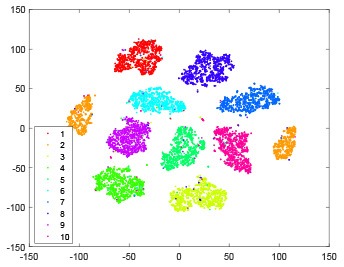
Output layer	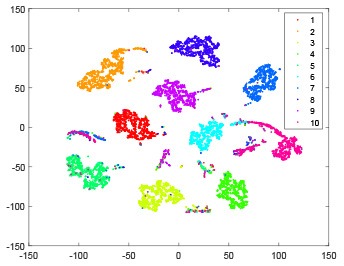	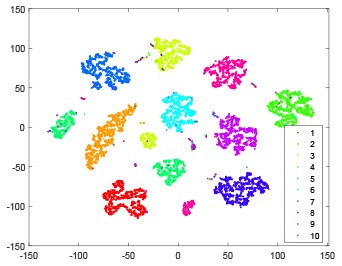	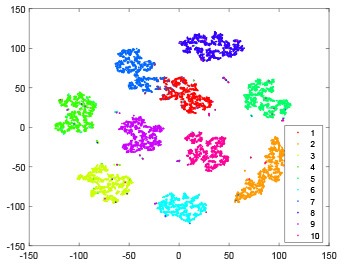	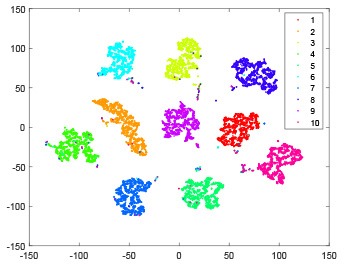
Computation ratio	0.92%	14.97%	27.88%	40.34%
Accuracy of CBSNN	0.9540	0.9813	0.9830	0.9836
Parameter setting two: hidden neurons = 400, *T*_*start*_ = 1, *NE*_*th*_ = 0.25, *I*_*re*_ = 50				
Hidden layer	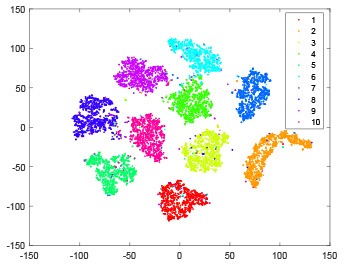	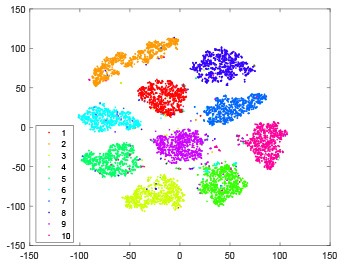	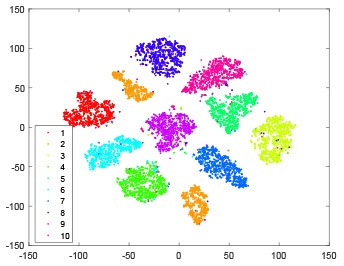	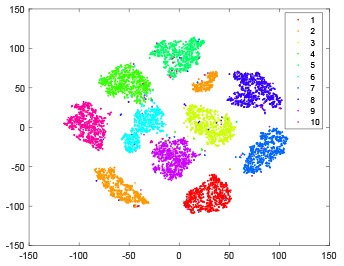
Output layer	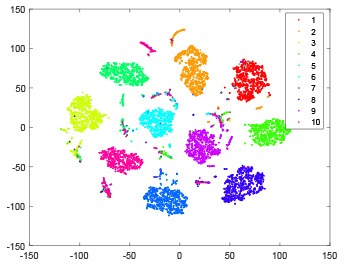	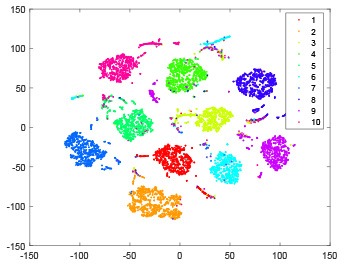	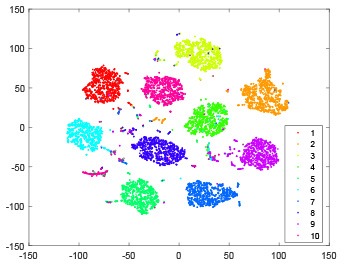	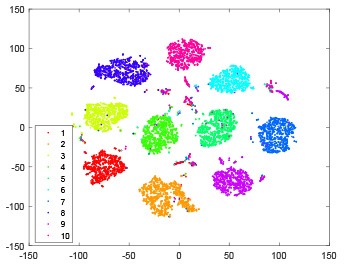
Computation ratio	1.24%	9.99%	15.12%	20.31%
Accuracy of CBSNN	0.9614	0.9708	0.9730	0.9753

*It shows the results of hidden layer, ouput layer, computation ratio (the baseline is VPSNN with 0.9826 accuracy and 100% computation cost) and accuracy of CBSNN after different iteration times*.

### 4.4. The Validation of CBSNN on Other Datasets

In this section, we will discuss how CBSNN performs in more applications. Here we adpoted Iris, NETtalk, Fashion-MNIST and CIFAR-10 datasets and in each task, CBSNN and VPSNN share the same network architecture. The corresponding results are shown in Table 3.

- Iris (Fisher, 1936) is a machine learning dataset for multiple variable analysis and contains 120 samples of three classes of Iris flower. We randomly separated it into 90 for training and 30 for test. Finally, CBSNN performs 100% classification accuracy with lower computation cost than VPSNN.
- NETtalk (Sejnowski and Rosenberg, 1987) is usually used for speech generation, consisting 5,033 training and 500 test. The input is a string of letters with fixed length of 7, which is encoded into 189 dimensions (each character has a 27 length one-hot vector). The output is 26 dimensions which represent 72 phonetic principles. For this mapping task with strong global regularities, VPSNN reaches 0.8680 accuracy. Although CBSNN is only slightly higher than VPSNN, it saves about half of the computation cost.
- Fashion-MNIST (Xiao et al., 2017) is more discrete and includes more semantic information than MNIST. It consists of 28*28 gray-scale images of 10 categories of objects in wearing, divided into 60,000 training samples and 10,000 test samples. From Table 3, CBSNN reaches the accuracy of 85.74% (higher than VPSNN 2.75%) with only 18.35% computation cost on it.
- CIFAR-10 (Krizhevsky and Hinton, 2009) contains 60,000 samples (50,000 for training and 10,000 for test) and has image size of 32*32 pixels with three channels, which will bring the growth of calculation exponentially and exceed the ability of these two networks. We used some dimension reduction methods for preprocessing it, i.e., RGBtoGray, Principal Components Analysis (PCA) and Convolution. From Table 3, the Convolution which converts the original data from 32*32*3 into 300 dimensions, works best and helps CBSNN achieve the best accuracy of 52.85% under only around a third of computation cost of VPSNN.

**Table 3:** The comparison of VPSNN and CBSNN on different tasks.

**Dataset**	**Preprocessing**	**Network architecture** **(input-hidden-output)**	**CBSNN** **Accuracy**	**VPSNN** **Accuracy**	**Computation ratio** **(based on VPSNN)**
Iris	None	4-2-3	**1.0000**	0.9667	64.59%
NETtalk	None	189-80-26	**0.8720**	0.8680	48.08%
Fashion-MNIST	None	784-400-10	**0.8574**	0.8299	18.35%
CIFAR-10	Gray	1024-500-10	**0.3198**	0.2947	71.69%
	PCA	700-400-10	**0.2546**	0.2384	15.51%
	Conv	300-100-10	**0.5285**	0.5134	31.11%

*In each task, CBSNN and VPSNN have the same network architecture and the accuracy of CBSNN (highlighted in Bold) is higher than that of VPSNN with lower computational cost (listed in the last column)*.

## 5. Discussion

SNN is the third-generation neural network (Maass, 1997). It has more biological structures and processing mechanisms, such as discrete sequential spike neurons which make it possible to deal with spatiotemporal information simultaneously, and non-BP biological plasticity like STDP. Although both SNN and ANN are black boxes at present, SNN has biological basis for reference but ANN does not, so there may be more applications of SNN in the future. At present, SNN has reached the accuracy comparable to that of deep network in many tasks, but it faces a serious problem: the time-consuming computation on neuron level and complex optimization limit their real-time application.

In this paper, we propose a CBSNN which is inspired by the curiosity-based learning mechanism in the brain. The CBSNN model can improve the accuracy and greatly reduce the computation of traditional SNN simultaneously. During the learning process, instead of feeding all data to the network, our model focuses more on mining difficult samples which is based on the novelty estimation. And in order to avoid the overfitting of the novel samples and forgetting of the learned samples, the CBSNN retrains all samples periodically. Finally, the CBSNN achieves comparable performance with the previous state-of-the-art VPSNN using just 54.95% computation of it. Similar conclusion can also be found out in other datasets, i.e., Iris, NETtalk, Fashion-MNIST, and CIFAR-10, respectively.

One of the main motivations of the paper is to dramatically decrease the training time of SNNs and make them better simulated on traditional computing systems by combining the biological plausible rules. Besides, with the development of neuroscience and physiology, more mechanisms in biological systems will be found out, which would further help SNNs on the faster processing speed and less computation cost.

## Data Availability Statement

Publicly available datasets were analyzed in this study. This data can be found here: http://yann.lecun.com/exdb/mnist/.

## Author Contributions

MS and YZ designed the study, performed the experiments and the analysis, and wrote the paper. YZ and TZ were involved in problem definition, algorithm discussion, and result analysis.

### Conflict of Interest

The authors declare that the research was conducted in the absence of any commercial or financial relationships that could be construed as a potential conflict of interest.
