# Corrigendum: A Curiosity-Based Learning Method for Spiking Neural Networks

**DOI:** 10.3389/fncom.2020.00028

**Published:** 2020-04-15

**Authors:** Mengting Shi, Tielin Zhang, Yi Zeng

**Affiliations:** ^1^Research Center for Brain-inspired Intelligence, Institute of Automation, Chinese Academy of Sciences, Beijing, China; ^2^University of Chinese Academy of Sciences, Beijing, China; ^3^Center for Excellence in Brain Science and Intelligence Technology, Chinese Academy of Sciences, Shanghai, China; ^4^National Laboratory of Pattern Recognition, Institute of Automation, Chinese Academy of Sciences, Beijing, China

**Keywords:** curiosity, spiking neural network, novelty, STDP, voltage-driven plasticity-centric SNN

In the original article, there was an error. In the original main text, there was an inaccurate statement sentence the result of NETalk in Table 3.

A correction has been made to ***Experiments***, ***The validation of CBSNN on other*** ***datasets***:

- NETtalk (Sejnowski and Rosenberg, 1987) is usually used for speech generation, consisting 5,033 training and 500 test. The input is a string of letters with fixed length of 7, which is encoded into 189 dimensions (each character has a 27 length one-hot vector). The output is 26 dimensions which represent 72 phonetic principles. For this mapping task with strong global regularities, VPSNN reaches 0.8680 accuracy. Although CBSNN is only slightly higher than VPSNN, it saves about half of the computation cost.

The authors apologize for this error and state that this does not change the scientific conclusions of the article in any way. The original article has been updated.

## References

[B1] Sejnowski T. J. Rosenberg C. R. (1987). Parallel networks that learn to pronounce english text. Compl. Syst. 1, 145–168.

